# Medical Items and News of Interest to the Physician

**Published:** 1869-04

**Authors:** 


### Of Interest to the Physician.

**Valuable Preparations of Glycerine.**

Four parts, by weight, of yolk of egg, to be rubbed in a mortar with five parts of glycerine. This compound has the consistency of honey, is unctious, like fatty substances, but is easily removed by water. Applied to the skin, it forms a varnish which effectually prevents the action of the air. It allays the itching in cutaneous affections. It is unalterable, and can be exposed to the air for an indefinite period.—*British Med. Journal, from Phil. Journal of Pharmacy.*

**Carbolate of Lime in Pertusses.**

Dr. Snow, Providence, R. I., has suggested the use of *carbolate of lime* in whooping cough, and in all cases it has apparently produced a marked effect in diminishing the frequency and severity of the paroxysms. Small quantities of the carbolate of lime are placed in saucers in the room where the child sleeps; merely sufficient to make the odor perceptible.

**Turpentine an Antidote to Phosphorus.**

It is stated in the *Archives Gen. de Medicine* that it is the custom of the workmen in the Stafford match factory, who apply phosphorus to the matches, to carry a tin cup on their breast, filled with essence of turpentine. The ill effects from the action of the phosphorus are thus prevented. It is extensively known that the vapor of turpentine prevents the ignition and phosphorescence of phosphorus.

**Epilepsy from Carious Teeth.**

Dr. Ramskill (*dental cosmos*) mentions the following case : A boy thirteen years of age, had frequent attacks of epilepsy, occurring about seven or eight o'clock in the evening. On examination a molar tooth was found considerably decayed, with swollen gum around it, and partly growing over into the cavity. It was not very tender to the touch, nor did the examination give rise to tooth-ache. The extraction of this tooth was followed by cessation of the fits.

**Amaurosis Caused by Crowding of Teeth.**

Mr. Hancock (*Lancet*) reports the following peculiar case: A boy, aged eleven, whose sight had been previously unimpaired, found upon waking one morning, that his sight was entirely lost. He was admitted to the Charing-Cross Hospital about a month afterwards, when it was found that his teeth were much crowded and wedged together; the jaws, in fact, not being large enough for them. Two permanent and four milk molar teeth were extracted, and the boy could distinguish light from darkness on the same evening; on the following morning he could make out objects. Eleven days after he was discharged cured, the only treatment beyond the removal of the teeth being two dose, of aperient medicine.

**Treatment of Boils, Whitlows, and Abscesses by Carbolic Acid.**

C. J. Clerborne, M. D.; Surgeon U. S. A., (*American Journal of Medical Science*) records his experience in the treatment of whitlows, boils and abscesses with this article. Being dissatisfied with the usual mode of treatment, he determined to try the effect of carbolic acid. This he did by making a free opening so soon as fluctuation could be detected, and when all the pus had been discharged by gentle pressure, the cavity was injected or swabbed out with the ordinary liquid carbolic acid of the shops, after which a cold water dressing was applied. In this way further suppuration was prevented, and the wound healed so rapidly that the patient returned to duty in two or three days. In some cases, after evacuating the pus, and using the acid, the edges of the wound were brought together with isinglass plaster, and in twenty-four hours it entirely healed.

**Poisoning from Eating Bread Containing Ergot.**

Dr. Flinzer (*Von Horn's Vjschr. f. Gess. Med.* VIII. 1868) relates that a whole family consisting of ten persons, after partaking of bread containing a large portion, say one-tenth, of ergoted rye, were taken seriously ill. Debility, giddiness, and loss of appetite were the first symptoms ; subsequently, tonic contraction of the extremities, a creeping sensation in the hands and feet, profuse perspiration, great thirst and diarrhœa were added. A pregnant female was brought to bed five weeks before her proper time. Two of the patients died ; but the fact became known too late to allow of an autopsy being made.

**An Antiseptic Sponge Tent.**

Dr. George Lyng Bryant, of Louisville, Ky., (*The Humboldt Med. Archives*) has prepared an *antiseptic* sponge as follows : He selects a moderately coarse, elastic sponge, which being well cleansed, while wet, cut into the shape and size required, is then saturated with thick gum mucilage, prepared by using ten or twelve grains of crystallized carbolic acid to the ounce, and wrapped on an awl, with a strong, well twisted cord. The tents should be fusiform, and wrapped from the small end, taking care that the layers of the cord are carried round in close proximity and with perfect regularity; and by retaining the "screw threads" thus formed, and turning it as an ordinary screw during insertion, the tent can be more easily introduced, and will not slip out as a smooth one is apt to do. granular erosions of the os and cervix uteri disappear in a short time by the use of this tent.

**Influence of Tobacco Upon the Circulatory System.**

Dr. Decaisne, in the course of investigation on the influence of tobacco on the circulation, has been struck with the large number of boys, aged from 9 to 15 years, who smoke, and has been led to inquire into the connection of this habit with impairment of the general health.

He has observed thirty-three boys, aged from 9 to 15, who smoked more or less. Of these, distinct symptoms were present in 27. In 22 there were various disorders of the circulation *bruit de souffle* in the neck, palpitation, disorders of digestion, slowness of intellect, and more or less marked taste for strong drinks. In three the pulse was intermittent. In eight there were found on examination more or less diminution of the red corpuscles; in twelve there was rather frequent epistaxis; ten had disturbed sleep, and four had slight ulcerations of the mucous membrane of the mouth, which disappeared on ceasing the use of tobacco for some days. In children, who were well nourished, the disorder was, in general, less marked. As to the ages, eight of the boys were from 9 to 12 years old; nineteeen 12 to 15. The duration of the habit of smoking was, in eleven, from six months to a year, and in fifteen, more than two years. Ordinary treatment of anæmia in general produced no effect as long as the smoking was continued; but when this was desisted from, health was soon perfectly restored, if there was no organic disease.

**Nasal Polypus.**

Dr. Gardner (*Boston Med. and Surg. Journal,*) reports a case of nasal polypus destroyed by the injection of persulphate of iron into its tissues.

**Trismus Neonatorum.**

Hot baths cause trismus neonatorum; consequently the physicians of Germany warn midwives not to make free use of them.

**Poisoning by Mushrooms.**

Dr. Poulet (Haute-Saone) has sent in a paper to the French Academy of Sciences to show that alcohol, taken in large doses, is a sure specific in cases of poisoning by mushrooms.

**Young Mother.**

Dr. King, of Rochford, Essex, states that he has attended a girl *eleven* years of age in confinement, and that mother and child are doing well.

**Treatment of Purpura.**

Dr. Neutershausen (Deutsche Klinik,) reports great success in the treatment of purpura hæmorrhagica with *secale cornutum.* He gives 8 to 10 grains three times, or oftener, daily, until hæmorrhagic manifestations cease. When anæmia remains he treats it with chalybeates.

**Opium and Stramonium.**

Dr. J. F. Treuman was called to see a mother and two daughters, who had accidentally taken a decoction of stramonium seed, instead of fennel seed. When first seen, they were raving like maniacs. Tr. opii was at once given, and morphia injected subcutaneously. They all recovered. So says the *Chicago Medical Journal.*

**Gonorrhœa.**

Finely powdered starch, mixed with lukewarm water, so as to obtain a fluid of the substance of cream, but thin enough to allow of injection, forms a most successful injection in cases of gonnorrhœa, when the inflammatory stage is over.

**To Prevent Death from Chloroform.**

Experiments on inferior animals show that they may be restored from apparent death by chloroform by the continuous galvanic current, the negative pole being placed in the mouth, and the positive pole in the rectum. In some cases the animal was left for two minutes in a state of apparent death, and then restored.—*Pacific Medical and Surgical Journal.*

**Cure for Nervous Headache.**

George Kennion, M. D., F. R. C. P., London, (*Med. Times and Gazette,*) advocates the following remedy for nervous headache. A small quantity of *bisulphide of carbon* (about ʒ-ij.) is poured upon the cotton wool, with which a small wide-mouthed glass bottle is half filled. The mouth of the bottle is then applied closely (so that none of the volatile vapor may escape)

to the temple, behind the ear, or as near as possible to the seat of pain; and there held for several minutes. After it has been applied for a minute or two, a sensation is felt as if several leeches were biting the part, and after the lapse of two or three minutes more, the smarting and pain become rather severe, but subside almost immediately after the removal of the bottle. Redness of the skin is seldom produced. The effect of this application is generally immediate; it may be reapplied, if necessary, three or four times a day.

**Malignant Pustule.**

Dr. Caspar in Stassfurth has used *liq. ammoniæ causticus* in several hundred cases, and reports that the only instance of death was that of a woman far advanced in pregnancy, who vomited the remedy as often as administered. He gives to children from one to three drops, to adults five drops, in sweetened oatmeal gruel, every hour by day and night. He applies dilute liq. chlorini to the ulcer, and continues the treatment until the inflammation in the neighborhood of the ulcer ceases to spread. —*Deutsche Klinik.*

**Prevention of Tubercle.**

Dr. McCormic, of Belfast, author of a treaty on consumption, says: "The result has been an intimate conviction that, *I have determined the real and only possible source of tubercle*, and to enhance and intensify the belief, that the profession, if they will only energetically and uniformly enforce the habitual respiration of air not pre-breathed, may within a very brief period, bring the ravages of tubercle to an absolute and permanent close."

### Cancer Quacks.

The *London Lancet* contains an account of the trial of a quack in Scotland for causing the death of a lady, by the application of an arsenic plaster to her breast, for the removal of some harmless excresence, which the quack frightened her into believing was cancerous.

The patient died in 10 days after the plaster was applied, and at the *post mortem* arsenic was found in the various tissues of the body, but no cancerous disease about the breast.

The quack was sentenced to imprisonment.—He should have been hung. We have no patience with these scalawages, much less with the people who employ them.

